# Supplementary material for: Time-restricted eating in early-stage Huntington’s disease: A 12-week interventional clinical trial protocol
Source: PLoS One. 2025 Mar 25;20(3):e0319253. doi: 10.1371/journal.pone.0319253 (PMC11936236; doi:10.1371/journal.pone.0319253)
Supplement: File S2 — (DOCX) [file pone.0319253.s002.docx]

1. Protocol Title

Time-Restricted Eating in Huntington’s Disease: A Clinical Pilot Study

2. Objectives

Huntington's disease (HD) is a devastating hereditary neurodegenerative disorder characterized by a triad of motor symptoms (including involuntary and bradykinetic movements), cognitive impairment, and psychiatric abnormalities that ultimately progress toward death over the course of 15 to 20 years from the time symptoms appear [1, 2]. Despite considerable efforts, effective disease-modifying treatments for HD remain elusive, necessitating exploration of novel therapeutic approaches, including lifestyle modifications that could delay symptom onset and slow disease progression. Growing evidence suggests that a form of intermittent fasting (IF) known as time-restricted eating (TRE) – defined by consuming all daily caloric intake within a 6-8 hour time window and fasting the remainder of the 24-hour day – may slow the progression of neurodegenerative diseases, including HD [3-6]. In this study, we will evaluate the feasibility, safety, and preliminary efficacy of TRE in persons with HD.

Several pre-clinical studies have revealed TRE’s potential to attenuate underlying pathologic mechanisms, mitigate disease severity, and prolong lifespan in animal models of HD [7-10]. TRE has also been associated with significant cognitive enhancements in non-HD elderly adults with mild cognitive impairment and has been used as a weight management strategy in overweight subjects [6, 11]. Safety, in this case, is a concern especially in a disease like HD known for causing weight loss. Although TRE has shown potential in animal studies and non-HD populations, it has yet to be analyzed for safety and feasibility in persons with HD. Given the existing evidence, we find it reasonable, applicable, and necessary to explore the application of TRE in persons with HD.

Specific Aim 1: Examine the feasibility and tolerability of TRE for treating HD.

Hypothesis 1: We hypothesize that at least 80% of participants will adhere to the TRE diet for the 12-week study duration with limited treatment-emergent adverse events.

Specific Aim 2: Evaluate the safety of TRE in early stages HD by measures of nutritional status.

Hypothesis 2: We hypothesize that TRE will have no negative impact on body weight, fat-free mass, vital signs, standard clinical markers of hematology, liver and kidney function, blood lipids, or fasting blood glucose.

Specific Aim 3: Analyze biomarkers of efficacy and explore whether TRE has beneficial effects on behavioral, cognitive, and motor function outcomes using standard HD clinical scales.

Hypothesis 3: We hypothesize that TRE will show trends toward improvements in biomarkers of HD progression and clinical measurements of these outcomes.

The aims will be accomplished by use of a 12-week prospective interventional open-label, single-arm trial that includes both field and in-laboratory data collection in persons with premanifest and early manifest HD. Specifically, participants will be asked to engage in a TRE diet, maintaining a 6-8-hour eating window every day for 13 weeks (+/- 1 week). A baseline (within 1-3 weeks of trial onset) and follow-up (within 7 days after week 12 of TRE) visit will assess body composition, safety labs, vital signs, clinical features of HD, and blood biomarkers. Participants will track the timing and content of diets, and record at-home body weight, physical activity, sleep, and mood using established survey techniques [11, 12].

*Primary endpoints: Feasibility, Safety, and Preliminary Efficacy*

Feasibility and tolerability: We expect that > 80% of subjects will adhere to the diet for the duration of the study with limited to no adverse events.

Body weight and fat-free mass: We expect that subjects will maintain their body weight and fat-free mass over the course of the 12-week study. Specifically, we hypothesize that at least 90% of subjects will have no more than a 10% loss in total body weight and no more than 5% loss in fat-free mass.

Vitals and blood chemistries: We expect that subjects will not experience adverse changes in vital signs, or measures of liver, kidney, or metabolic function.

Plasma neurofilament light protein (NfL) and Glial fibrillary acidic protein (GFAP): We expect that there will be an improvement in measures of plasma NfL and GFAP from baseline to follow-up.

*Secondary endpoints: Clinical Efficacy and Mechanism of Action*

Unified Huntington’s Disease Rating Scale (UHDRS) clinical assessment: We expect to see minor improvements in UHDRS motor, cognitive, independence, and functional capacity assessments; however, given the short-term nature of the study, it is unlikely significant changes will be observed.

Biomarkers of HD progression and TRE mechanism of action: We hypothesize that we will see beneficial changes in plasma markers of neurodegeneration (NfL and GFAP) and measures of cellular bioenergetics (mitochondrial function), but we have limited data at this time to support this hypothesis.

3. Background

Evidence suggests the mutant huntingtin protein (*mHtt*) found in HD induces neuronal dysfunction by disrupting various cellular components and pathways resulting in oxidative damage, transcriptional dysregulation, excitotoxicity, and bioenergetic deficiencies [13-15]. As the disease progresses, areas outside the brain become affected, leading to atrophy of skeletal muscle and dysfunction in the circadian rhythm [16, 17]. Incomplete understanding of the precise functions of wild type *Htt* paired with the heterogeneous harmful effects caused by *mHtt* has made the underlying pathology difficult to target therapeutically. For example, there is a well-known connection between CAG repeat length and age at disease onset at the population level, yet there is much variability in individual symptom timing, progression, and severity [18, 19]. Genome-wide association studies have highlighted DNA repair mechanisms, mitochondrial redox factors, and CAA alleles as contributors to this variability; however, environmental factors and lifestyle habits are also thought to modify age of onset and severity of disease [20-23]. Identifying and addressing these lifestyle habits could hold therapeutic potential in delaying HD progression.

Oftentimes pharmacological interventions target a very specific aspect of disease or physiologic process. Because *mHtt* has effects on so many areas of function, lifestyle interventions that can affect multiple processes and pathways may be more beneficial and are worthy of investigation. Growing evidence suggests that TRE may have the potential to attenuate the progression of neurodegenerative diseases [3-6]. TRE may be particularly well-suited for use in the HD population because of its ability to induce autophagy and clear *mHtt,* upregulate cytoprotective genes and brain derived neurotrophic factor (BDNF) production, improve mitochondrial bioenergetics and prevent oxidative stress, and regulate circadian function. Studies of TRE in HD transgenic animal models have found it increases *mHtt* clearance in the brain and attenuates the formation of huntingtin inclusions, motor dysfunction, glucose intolerance, and tissue wasting, all while extending animal lifespan and improving circadian rhythm synchronization [7-10]. Although there is very little experience with TRE in HD, a case study in a 41-year-old male HD patient with progressively deteriorating symptoms, reported that a 48-week combined metabolic strategy of TRE paired with a ketogenic diet resulted in improved motor symptoms, activities of daily living, composite Unified HD Rating Scale (UHDRS) score, and psychiatric symptoms [24]. In a 36-month longitudinal study consisting of 99 elderly subjects with mild cognitive impairment, consistent adherence to a TRE diet was associated with significant cognitive enhancements, decreased markers of DNA damage and inflammation, and improved markers in oxidative stress [6]. Because TRE diets have been applied as a weight management strategy in overweight individuals, safety is a primary concern as HD is known for causing weight loss, especially in later disease stages [11, 25]. It is interesting and reassuring that a study evaluating the safety and tolerability of TRE in healthy midlife and older adults found that the diet had no influence on body mass, fat-free mass, bone density, or nutrient intake [12].

Although TRE has shown potential in animal studies and non-HD populations, it has yet to be analyzed for safety, feasibility, or efficacy in the clinical setting. To address this clinical relevance and lack of existing knowledge, we will perform an interventional study in patients with premanifest and early manifest HD to examine if a TRE diet appears safe and explore efficacy regarding HD symptoms and biomarkers of disease progression. Our ultimate goal is to look at TRE in HD on a larger and longer scale. The rationale for this study is grounded in the desperate need to discover therapeutic measures that can reshape the course of HD progression and existing evidence of a simple lifestyle change that may have the potential to do so. It is also critical to discourage such practices if we see negative consequences of TRE. Using what is known of the TRE mechanism of action and current data, we expect that the diet will be safe, feasible, and may also improve biomarkers of disease progression and motor, cognitive, and behavioral function in patients with manifest HD. If this is true, our study will lay the foundation for future large-scale clinical trials to prove the efficacy of TRE in a life-threatening neurodegenerative disease.

4. Study Design

The primary aim of this study is to assess the feasibility, safety, and preliminary biomarker efficacy of a TRE diet in persons with HD. We will also explore clinical efficacy and potential mechanism of action. The proposed study will be a prospective interventional, open-label, single-arm trial (Fig 1). After baseline testing and a 1-3 week lead-in period, enrolled participants will be asked to engage in a TRE diet, specifically maintaining a 6-8-hour eating window every day for 13 weeks (+/- 1 week). Participants will be allowed to self-select the timing of the eating window, but once selected, they will be asked to maintain that schedule at minimum 5 of 7 days for the duration of the study. Outside of that window, for the remaining 16-18 hours of day/night, participants will be asked not to consume calorie-containing food or drink. Beverages without calories will be allowed. We will measure body weight and composition, safety labs, adherence to the diet, dietary composition, sleep, physical activity, mood, and markers of efficacy. If our primary measure of safety, as represented by stable body weight and fat-free mass, is not maintained, the study will be halted immediately. Data collection episodes will take place at the OHSU Department of Neurology outpatient clinic area within 1-3 weeks prior to the start of the study, and again within 7 days after 12 weeks of TRE. Participants will complete study surveys directly in Qualtrics. Survey invitations and reminders will be sent by email or SMS text message to study participants. A maximum of 3 reminders will be sent to a participant for any incomplete form. Participants will be contacted via phone call if there are 2 or more days without completing meal tracking surveys.

1. Safety, Biomarkers, and Clinical Efficacy: At baseline (within 1-3 weeks of trial onset) and follow-up (within 7 days after week 12 of TRE) participants will visit the OHSU Department of Neurology clinic for body weight assessment and body composition analysis via bioelectrical impedance analysis. Vitals will be measured by a trained researcher and blood will be drawn to obtain safety labs (chemistry and hematology panel, lipids, A1c, blood glucose, and creatinine clearance) and blood samples for biomarker assessments of efficacy. Participants will then be assessed by a trained medical professional using the Unified Huntington’s Disease Rating Scale (UHDRS).
2. Diet Adherence and Sleep: A daily electronic survey will be sent to each participant via text message or email, depending on participant preference, in the evening at 19:00 h. Participants will be asked to report the time of day they started and stopped eating, and their sleep habits and sleep quality from the previous night.
3. Dietary Composition: Twice per week, participants will be asked to use the SnapCalorie^TM^ phone application to record the meals eaten in a given day. At the baseline visit, participants will be instructed how to download and use the phone application. A text or email reminder will be sent in the morning at 07:00 h on the days that the participants are asked to record their meals.
4. Weekly Survey: Participants will be asked to complete weekly surveys to measure self-reported body weight, mood, and physical activity. The survey will be completed via Qualtrics. Researchers will send reminder text messages each week with a link to the relevant survey.
5. Safety and Tolerability: At the baseline visit, participants will be given a scale to measure at-home body weight. Every other week during the 12-week study, participants will be contacted by telephone to assess at-home body weight and discuss adherence issues or any adverse events. If a participant loses more than 10% of their total body weight, they will be asked to stop the TRE diet and return to their prior dietary habits.


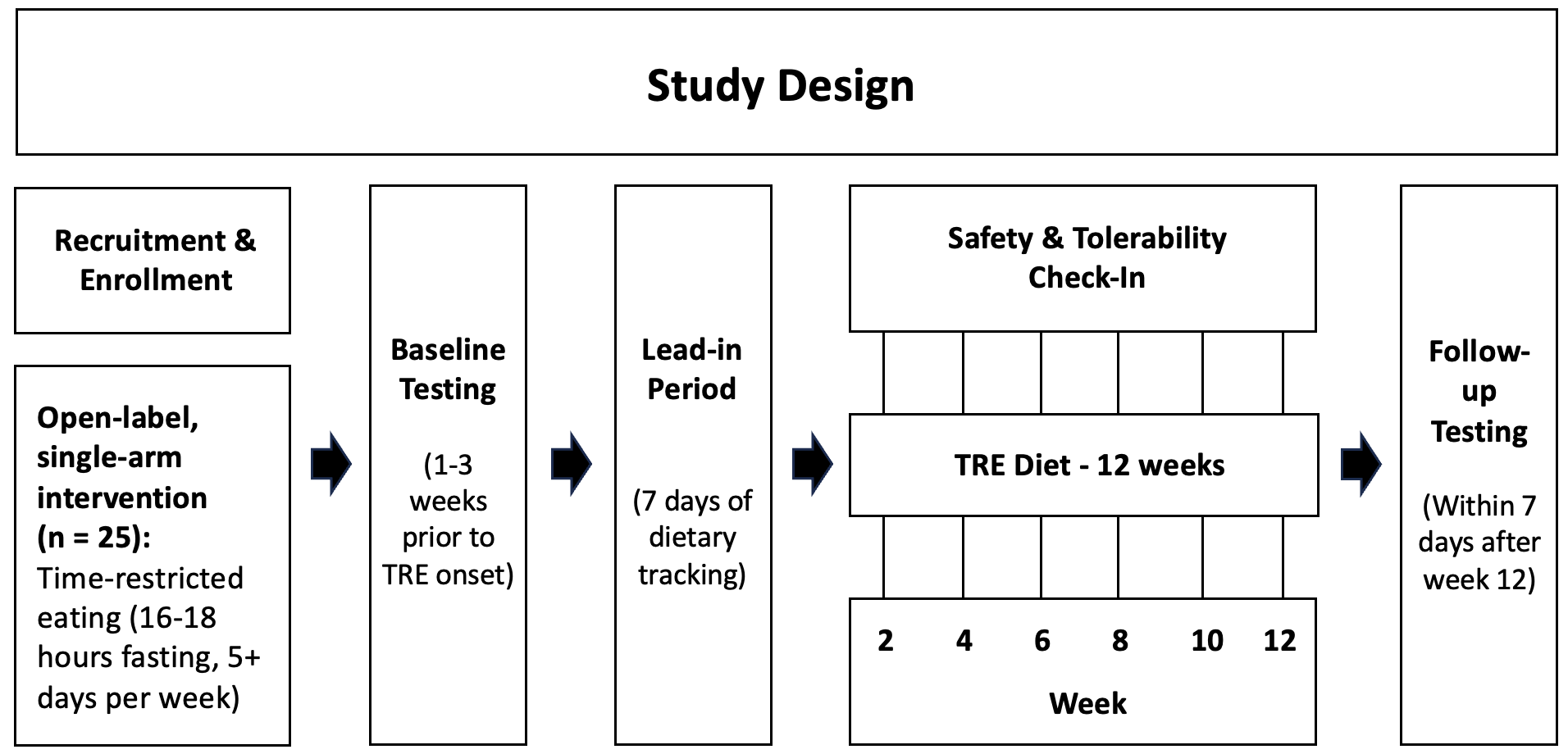


**Figure 1**. TRE in HD Study Design

5. Study Population

1. Number of Subjects

We will recruit 40 participants expecting roughly 25 participants to meet inclusion criteria with the goal of yielding a final sample of at least 20 in the experimental condition after a conservative expected level of attrition (~20%). These individuals will consist of males and females 21 years of age or older, with genetically confirmed premanifest or early manifest (stage I and II) HD defined by a CAP score greater than 368 or UHDRS TFC greater than or equal to 7, respectively. Subjects will be recruited from Oregon and Washington. See Statistical Analysis Section for justification of the selected sample size.

1. Inclusion and Exclusion Criteria

Inclusion Criteria

Subjects eligible to participate in this study are persons who:

1. Are of at least 21 years of age at Screening.

2. Must fulfill one of the following criteria:

a. Premanifest late prodromal HD as defined by a genetically confirmed CAG repeat ≥ 36 and a CAG-Age Product (CAP) score > 368 (CAP = (Age) x (CAG – 33.66)).

b. Early manifest (stage I and II) HD as defined by a TFC greater than or equal to 7. Subjects must have been determined to have a clinical diagnosis of HD by the site investigator as defined by a diagnostic confidence level (DCL) of 4.

3. Must fulfill both of the following criteria:

a. Have undergone genetic testing with a known CAG repeat greater than or equal to 36.

b. No features of juvenile HD (Westphal variant)

Clarification of CAG Repeat Number (Allele length) Testing Requirements:

A CAG repeat number obtained prior to the Screening Visit will be used to

document subject eligibility if at Screening there is documentation available in

the subject’s record that states that the subject has an expanded CAG repeat (greater than or equal to 36) from a prior validated laboratory assessment.

4. All female subjects of childbearing potential must have a negative urine pregnancy test at baseline, and female subjects of childbearing potential must practice a highly effective method of contraception (e.g., oral contraceptives, a barrier method of birth control [e.g. condoms with

contraceptive foams, diaphragms with contraceptive jelly], intrauterine devices, partner with vasectomy or sexual abstinence) for the duration of the study.

5. Are willing and capable of providing informed consent for study participation.

6. Are capable of reading, writing, and communicating effectively with others.

*Exclusion Criteria*

Subjects ineligible to participate in this study are persons who:

1. Have participated in an investigational drug or device study within 30 days of the baseline visit

2. Have had previous neurosurgery for Huntington’s disease or other movement disorders.

4. Have clinically significant cognitive impairment that hinders the ability to appropriately consent or adhere to detailed study directions, in the opinion of the principal investigator.

5. Have a presence of clinically significant psychosis and/or confusional states, in the opinion of the site Investigator.

6. Have clinically relevant hematologic, hepatic, cardiac, thyroid, or renal disease.

7. Have a history of substance abuse (based on DSMIV criteria) within the past 12 months prior to screening.

8. If female, are pregnant or breastfeeding.

9. Have a high-risk for nutritional deficiency.

10. Are not weight stable for at least three months prior to enrolling in the study, defined as greater than 2 kg change in body mass.

11. Express a desire to lose weight during the study.

12. Have a clinically significant medical, surgical, laboratory, or behavioral abnormality which in the judgment of the site Investigator makes the subject unsuitable for the study.

13. Have consistently practiced a time-restricted eating protocol within 3 months of trial onset.

In the event of a screen failure, data acquired will be destroyed immediately.

1. Vulnerable Populations

Persons with HD are a potentially vulnerable population; however, the focus on premanifest and stage I/II disease and cognitive requirements for study inclusion should limit the probability of working with those that are particularly vulnerable.

1. Setting

All research will be performed at OHSU and the research facilities at OCTRI.

1. Recruitment Methods

Subjects will be recruited through the OHSU Movement Disorders clinic patient pool using a combination of approaches, including clinician recruitment and referral during clinic appointments, matching from an IRB approved research registry (OHSU IRB #8049), advertising via an IRB approved flyer, and contacting participants of Enroll-HD (OHSU IRB #21855). Potential participants will be contacted either directly in clinic or by phone, utilizing an IRB-approved phone script.

Based on both recruitment methods and knowledge of the OHSU Movements Disorders HD patient population demographics, we do not anticipate identifying or enrolling any non-English speaking participants. However, if a participant is identified, study staff will obtain translation of the consent form, and will utilize translation services to conduct the study surveys verbally. We would not be able to obtain data via the SnapCalorie^TM^ app, as it is not available in languages other than English.

Participants will be paid for their participation in the study, up to $75 total if they complete the entire study -- $25 for completion of the Baseline visit, $25 for completion of the Follow-up Visit, and $25 as a Study Completion Bonus. If participants do not complete the study, they will be paid for the visits they do complete.

1. Consent Process

Potential participants will first be contacted by phone and study staff will utilize an IRB-approved phone script to assess initial interest and eligibility for the study. Those who remain interested and eligible will be provided the consent form for review. The potential participant will be contacted by phone a few days later and if still interested, will be scheduled for a baseline visit in clinic. At the baseline visit, investigators will go over each aspect of the consent form in plain English and answer any questions the participants may have. When the participant is satisfied with his/her understanding of the study, and fully comprehends his/her freedom to withdraw at any time, the potential participant will be asked to sign the paper research consent form if they wish to do so.

The investigators will take all necessary steps to answer questions raised by volunteers pertaining to the nature, purpose and risks of the study. An investigator will be available at all times to answer questions the participant may have. By both verbal and written instruction, the participant will be explicitly informed of his/her freedom to withdraw consent and discontinue participation in the project at any time.

6. Procedures Involved

1. **Baseline and Follow-up Visits:** 1-3 weeks prior to the onset of the study and after week 12 of TRE, participants will visit OHSU where the following procedures will take place:

*Body Weight and Composition:* A trained researcher will collect body weight and measures of body composition (fat-free mass, fat mass, bone mass) using a validated bioelectrical impedance (BEI) scale in OCTRI’s Clinical and Translational Research Center (CTRC).

*Vital Signs:* A trained researcher in the same clinic space will record participant body temperature, pulse rate, respiration rate, and resting blood pressure. Blood pressure will be measured using a calibrated instrument after 5 minutes of quiet sitting using an appropriately sized arm cuff.

*Safety Labs and Biomarker Samples:* Chemistry and hematology panels, lipids, A1c, blood glucose, and creatinine clearance labs will be obtained via venous blood samples that will be collected to further measure the safety profile of the intervention. Additional blood will be drawn for biomarker analysis to assess intervention efficacy and exploratory effects (Table 2). A total of 60 mL of blood will be drawn. These samples will be collected while fasting by a certified phlebotomist in the OCTRI CTRC outpatient clinic.

*In-Lab Demographics and Health Behaviors Surveys:* At baseline, participants will complete a demographics and health behavior survey and at follow-up they will complete a shorter version of the same health behaviors survey.

*Montreal Cognitive Assessment:* A trained researcher will administer the Montreal Cognitive Assessment (MoCA), a validated test used to measure cognitive function, at the baseline and follow-up visits.

*Unified Huntington’s Disease Rating Scale (UHDRS):* The UHDRS is a clinical rating scale developed to assess four domains of HD – motor function, cognitive function, behavioral abnormalities, and functional capacity. The scale will be used to track and assess for clinical effects of TRE. The assessment consists of four parts with separate scoring for each part:

Part I: Motor function. 31 items with 5-point ordinal scale ranging from 0-4 that indicates performance on motor tasks.

Part II: Cognitive function. Three items including the Verbal Fluency Test, Symbol Digit Modalities Test, and the Stroop Interference Test that evaluate cognitive performance.

Part III: Behavioral assessment. 10 items with a 5-point ordinal scale ranging from 0-4 that indicates the severity of behavioral symptoms associated with HD.

Part IV: Functional capacity. This domain is divided into three sections:

1. Huntington’s Disease Functional Capacity Scale (HDFCS) is reported as the Total Functional Capacity Score (TFC) which has a total of 25 yes/no questions assessing the total functional capacity of the individual.
2. Independence Scale rated from 10-100 indicating functional level of independence.
3. Functional capacity assessment consisting of five items with a 4-point ordinal scale ranging from 0-4.

A trained medical professional will complete the total UHDRS with each participant in the OCTRI CTRC outpatient clinic.

1. **Lead-in Period:** During the 1-3 week lead-in period after the baseline visit, participants will be asked to use the SnapCalorie^TM^ phone application and to complete the daily meal timing and sleep survey to log at least 7 days of their normal dietary and sleep habits. Daily calories and macronutrients will be calculated and used to provide dietary caloric goals during the 12-week TRE intervention.
2. **Time-Restricted Eating Protocol:** During the baseline visit, participants will meet with researchers and be informed of the intervention. They will be instructed to maintain a 6-8-hour eating window every day for 12 weeks after completion of the lead-in period. Participants will be allowed to self-select the timing of the eating window, but once selected, should attempt to maintain the schedule at a minimum 5 of 7 days per week for the duration of the study. Outside of that window, for the remaining 16-18 hours of day/night, participants will be asked not to consume calorie-containing food or drink. Beverages without calories, such as black coffee or tea, will be allowed. Participants will be instructed to consume plenty of water and electrolytes. Participants will also be provided a document with information about general healthy diet and exercise habits, in addition to instructions about the TRE protocol and a brief overview of each study visit..
3. **Daily Participant Tasks:**

*Meal Timing and Sleep Survey:* A daily electronic survey will be sent to each participant via text message or email, depending on participant preference, in the evening at 19:00 h. Participants will be asked to report the time of day they started and stopped eating, and their sleep habits and sleep quality from the previous night. Specifically, they will be asked to report their sleep duration, quality, and number/length of naps that day.

1. **Weekly Participant Tasks**:

*Dietary Content Assessment:* Twice per week, participants will be asked to use the SnapCalorie^TM^ phone application to capture the meals eaten in a given day. At the baseline visit, participants will be instructed how to download and use the phone application. A text or email reminder will be sent in the morning at 08:00 h on the days that the participants are asked to record their meals. Using the application, participants will be asked to take photos of each meal and report estimated serving sizes. The application uses artificial intelligence technology to analyze meal composition which will be specifically used to estimate caloric content, and daily consumed fats, carbohydrates, and proteins.

*Weekly Survey:* Participants will be asked to complete a weekly survey to measure self-reported body weight, mood, and physical activity. The survey will be completed via Qualtrics. Researchers will send a reminder text or email each week with a link to the survey.

1. **Alternate Week Check-In:**

*Body Weight and Adverse Events Phone Call:* At the baseline visit, participants will be given a scale to measure at-home body weight. Every other week during the 12-week study, participants will be contacted by telephone to discuss at-home body weight changes and any adherence issues or adverse events.

Table 1. Study Visits and Detailed Procedures

|  |  | **Study Schedule** | | | | | | | | |
| --- | --- | --- | --- | --- | --- | --- | --- | --- | --- | --- |
| **Procedures** | | | **Pre-visit** | **Baseline Visit** | **Lead-in** | **Daily** | **Weekly** | **Weeks 2,4,6,8,10,12** | **Follow-up Visit** |  |
| Telephone Screening, Eligibility, and Consent | | | X |  |  |  |  |  |  |  |
| Survey Screening, Eligibility, and Consent | | | X |  |  |  |  |  |  |  |
| Montreal Cognitive Assessment | | |  | X |  |  |  |  | X |  |
| Consent | | |  | X |  |  |  |  |  |  |
| Pregnancy Test | | |  | X |  |  |  |  |  |  |
| In-Lab Questionnaire | | |  | X |  |  |  |  | X |  |
| Weight and Body Composition via BEI | | |  | X |  |  |  |  | X |  |
| Vital Signs | | |  | X |  |  |  |  | X |  |
| Venipuncture Blood Draw | | |  | X |  |  |  |  | X |  |
| UHDRS Assessment | | |  | X |  |  |  |  | X |  |
| Meal Timing and Sleep Survey | | |  |  | X | X |  |  |  |  |
| SnapCalorie^TM^ Meal Tracking | | |  |  | X |  | X |  |  |  |
| Body Weight, Mood, and Physical Activity Survey | | |  | X |  |  | X |  |  |  |
| Telephone Check-In | | |  |  |  |  |  | X |  |  |
| Total Blood Draw Estimates | | |  | 30 mL |  |  |  |  | 30 mL |  |
| Time Estimate | | | 5 min | ~2h | ~10 min | ~5-min | ~30-min | ~5-min | ~2-h |  |

**Table 2. Biomarker Analysis**

| **Primary Outcome Measures** | | | | |
| --- | --- | --- | --- | --- |
| **Biomarker** | **Definition** | **Measurement** | **Indication for Use** | **Method** |
| Neurofilament light protein (NfL) | A protein component of the neuronal cytoskeleton released into the CSF and blood from damaged neurons | A serum biomarker of neurodegeneration in HD, correlates with disease onset, stage, severity, and progression [26, 27] | Proof-of-concept measure of efficacy | ELISA or Single-molecule array (SIMOA) |
| Glial fibrillary acidic protein (GFAP) | A protein of astrocytes, increased in astroglial activation | A serum biomarker of neurodegeneration in HD, correlates with NfL, disease stage, and progression [28] | Proof-of-concept measure of efficacy | ELISA or Single-molecule array (SIMOA) |
| **Secondary Outcome Measures** | | | | |
| Peripheral mutant huntingtin protein (mHTT) | A protein known to play a critical role in the pathogenesis of HD | A leukocyte biomarker that correlates with HD severity, onset, and progression [29-31] | Exploratory mechanism of action measure | ELISA-based MSD |
| ULK1 | A mammalian serine/threonine protein kinase, plays a key role in the initial stages of autophagy | mRNA expression of ULK1 correlates with autophagic activity, potentially upregulated by TRE [32, 33] | Exploratory mechanism of action measure | qPCR |
| NRF2 | A transcription factor that regulates endogenous antioxidant response pathways | mRNA expression of NRF2 correlates with ROS response, upregulation has therapeutic indications in HD, potentially upregulated by TRE [34-36] | Exploratory mechanism of action measure | qPCR |
| Bioenergetic profile | A description of cellular energy metabolism characteristics | A measure of mitochondrial function and ETC activity, altered in HD, and potentially improved by TRE [37] | Exploratory mechanism of action measure | Seahorse analyzer |

7. Data and Specimens

- 1. Handling of Data and Specimens
     1. **Electronic Data Handling**: While the study is active, all PHI will be stored in a database housed on a secure OHSU server. PHI will be accessible to only investigators, and research staff listed on the protocol. No PHI will be stored or transferred via USB or other portable drives. We will obtain a waiver of the HIPPA authorization requirement for PHI collect at the time of the phone and survey screen.
        1. Research Electronic Data Capture (REDCap) and Qualtrics: Data and recruitment information for this project will be stored in OCTRI's installation of REDCap or Qualtrics, two highly secure and robust web-based research data collection and management systems. Features of REDCap that protect participants' privacy and data security include:

1. Physical Security: OCTRI's REDCap software is housed on servers located in ITG's Advanced Computing Center providing locked physical security
2. Electronic Security: The REDCap servers are housed behind both the OHSU firewall and a second ACC firewall.  All web-based data transmissions are encrypted with industry-standard SSL methods.
3. Controlled User Access: REDCap is employs a robust multi-level security system that enables researchers to easily implement "minimum necessary" data access for their research staff, including specification of data fields that are identifiers. This feature includes “single click” ability to provide completely deidentified (removing all identified data fields and shifting dates) for analysis or other purposes.  User activities are logged to enable auditing of all data access.   Access is integrated with OHSU's network such that users who are also OHSU employees are authenticated against their OHSU network credentials.
4. Data Integrity: REDCap is jointly managed in accordance with OHSU Information Security Directives by ACC staff and members of OCTRI's Biomedical Informatics Program, ensuring fidelity of database configuration and back-ups.  User activities are logged to enable auditing of all data changes.
   - 1. At the initial phone-screen, all participants will be assigned a unique code that will be used to identify them on documents residing outside of the secure database. Upon enrollment, participant study data will be recorded and stored in a password protected RedCap or Qualtrics database and will be identified only by the unique code assigned at the initial phone screen. Only persons listed on the IRB approved protocol will be given access to the databases.
     2. **Paper Data Handling:** All paper files (e.g. signed consent forms, participant sleep and meal timing diaries) will be stored in locked filing cabinets in restricted access offices at OHSU. Original records will be retained as the source document. Access to these files will be limited to study personnel listed on the IRB approved protocol.
     3. **Specimen Handling:** Blood samples will be identified only by the code assigned at the initial phone screen. Processing and handling of blood samples will be done by the OCTRI nursing and core lab staff. Samples will be stored and maintained by the Quinn Lab -80 degree freezer. Access to study samples at OHSU will be limited to OCTRI core lab staff and study personnel listed on the IRB approved protocol. Coded biological samples may be sent to the Quanterix Corporation Laboratory for analysis.
   1. Sharing of Results with Subjects

With their consent, we will make known to the participant some of the information we have gathered from the physiological testing during the study. There is a chance that the study assessments and/or lab results will reveal some medical abnormality. This information will be conveyed to the participant, together with a recommendation to discuss the results with a physician if appropriate. All blood data collected and shared with participants is from a CLIA certified lab (OHSU).

- 1. Data and Specimen Banking

Venous blood samples will be stored for future research, which may include genetic research.

Samples will be hand carried by the study staff to the Hatfield Research Center and stored in secure freezers room for storage. Any remaining specimens after the required analyses have been conducted will be stored securely in the Hatfield Research Center. Samples may be shared with other investigators at OHSU or investigators outside of OHSU. All appropriate and required agreements will be in place prior to the release of samples. Samples will be identified by an assigned study participant ID number and will be transported by an approved courier. For any future use of these samples, IRB approval must be obtained. The repository guardian will be Dr. Amie Hiller, who will be responsible for managing future access to these samples.

8. Data Analysis

The primary goals of this study are to assess if TRE in persons with premanifest and early manifest HD is 1) feasible, 2) safe, and 3) potentially efficacious.

Specific Aim 1: Examine the feasibility and tolerability of TRE for treating HD.

The percent of adherent days to the TRE intervention will be calculated and any adverse events will be recorded and described in detail.

Specific Aim 2: Evaluate the safety of TRE in premanifest and early manifest HD by measures of nutritional status.

The primary safety outcome will be percent change in body weight and fat-free mass which will be examined using planned comparison paired t-tests between the baseline and follow-up visits. Differences in measures of vital signs, and safety labs will also be examined using planned comparison paired t-tests between the baseline and follow-up visits as additional safety measures.

Specific Aim 3: Explore whether TRE has beneficial effects on biomarkers of disease progression and behavioral, cognitive, and motor function outcomes using standard HD scales.

Differences in measures of blood biomarkers and UHDRS clinical scores will be examined using planned comparison paired t-tests between the baseline and follow-up visits.

Table 3. Statistical Analysis

| Measure | Statistical Test (for each measure) |
| --- | --- |
| Daily eating period (length of time) | Paired t-test from Baseline to Follow-up |
| Adherent days | Percent of days adherent to the TRE diet |
| Adverse events | Descriptive statistics |
| Body weight | Paired t-test from Baseline to Follow-up |
| Fat-free mass | Paired t-test from Baseline to Follow-up |
| Vital signs (Temp, BP, HR, RR, O2) | Paired t-test from Baseline to Follow-up |
| Safety labs (LFTs, Cr and BUN, CMP, A1c, BG, Lipids) | Paired t-test from Baseline to Follow-up |
| Biomarkers (NfL and GFAP) | Paired t-test from Baseline to Follow-up |
| UHDRS | Paired t-test from Baseline to Follow-up |
| Dietary composition | Descriptive statistics |
| Self-reported sleep habits | Descriptive statistics |
| Self-reported physical activity | Descriptive statistics |
| Mood | Descriptive statistics |

1. Sample Size, Expected Outcomes, and Translation of Results

Aside from a single patient case report, an examination of TRE in persons with HD has never been explored. Thus, we must rely on existing data on TRE intervention in other populations. To detect differences in the percent change in body weight (Specific Aim 2) from baseline to follow-up, assuming a standard deviation of 2.4% from Gabel et al., we will have 95% power to detect a difference as low as 2.5% change in body weight in n=15 with two-sided Type I error of 0.05. To detect differences in plasma NfL (Specific Aim 3) from baseline to follow-up, assuming a standard deviation of 0.65 log pg/ml from Byrne et al., we will have 90% power to detect a difference as low as 15% in plasma NfL between baseline and follow-up in n=20 with two-sided Type I error of 0.05. *Thus, we will* *study 20 participants to maximize power while balancing feasibility* to derive meaningful proof-of-concept data.

9. Privacy, Confidentiality, and Data Security

Study staff will prepare and maintain complete and accurate study documentation in compliance with good clinical practice standards and applicable federal, state and local laws, rules and regulations. Specimens collected will include only a subject ID number and collection date on the collection and aliquot tubes. Data for this project, including demographics, medical and medication history, cognitive test results, and lab results will be stored in OCTRI's installation of REDCap, a highly secure and robust web-based research data collection and management system. Only researchers, including investigators and study coordinators, involved in this protocol will have access to the REDCap data. Paper files will be stored in restricted access offices in Sam Jackson Hall at OHSU, in locked offices contained within a key-code entry office suite.

10. Provisions to Monitor the Data to Ensure the Safety of Subjects

See *Data and Safety Monitoring Plan.*

11. Risks and Benefits

1. Risks to Subjects

*TRE Diet:* It is possible that participants will lose a small percent of body weight (not expected to be more than 2.5%) and it is expected to be primarily fat-mass. Participants may feel hungry during the fasting period and will be advised to stay properly hydrated to avoid feeling weak or light-headed.

*UHDRS Assessment:* The UHDRS clinical assessment requires attention and physical effort. Participants may experience minimal mental and/or physical fatigue.

*SnapCalorie^TM^ Phone Application:* The phone application participants will be asked to use twice per week is a commercially available service and we cannot guarantee data privacy or security through its use; however, personal information is not required and the only data that will be collected is of meal composition.

*Blood Draws:* There may be some discomfort or bruising on initial insertion of the needle into a vein.

*Survey Data:* Participants may be uncomfortable or feel concerned about answering survey questions about their personal sleep, mood, and health conditions. Surveys from this protocol may also not be generalizable outside of the English-speaking population.

1. Potential Benefits to Subjects

Although there will be no direct known physical benefit resulting from participation in this study, we will make known to the participant some of the information we have gathered throughout the study. There is a chance that the various samples taken during the study will reveal some medical abnormality. This information will be conveyed to the participant, together with a recommendation of a local clinic or physician from whom to seek treatment.

References

[1] Vonsattel JP, DiFiglia M. Huntington disease. J Neuropathol Exp Neurol. 1998;57(5):369-84 doi: 10.1097/00005072-199805000-00001.

[2] Solberg OK, Filkuková P, Frich JC, Feragen KJB. Age at Death and Causes of Death in Patients with Huntington Disease in Norway in 1986-2015. J Huntingtons Dis. 2018;7(1):77-86 doi: 10.3233/jhd-170270.

[3] Anton S, Ezzati A, Witt D, McLaren C, Vial P. The effects of intermittent fasting regimens in middle-age and older adults: Current state of evidence. Exp Gerontol. 2021;156:111617 doi: 10.1016/j.exger.2021.111617.

[4] Hadem IKH, Majaw T, Kharbuli B, Sharma R. Beneficial effects of dietary restriction in aging brain. J Chem Neuroanat. 2019;95:123-33 doi: 10.1016/j.jchemneu.2017.10.001.

[5] Fontana L, Ghezzi L, Cross AH, Piccio L. Effects of dietary restriction on neuroinflammation in neurodegenerative diseases. J Exp Med. 2021;218(2) doi: 10.1084/jem.20190086.

[6] Ooi TC, Meramat A, Rajab NF, Shahar S, Ismail IS, Azam AA, Sharif R. Intermittent Fasting Enhanced the Cognitive Function in Older Adults with Mild Cognitive Impairment by Inducing Biochemical and Metabolic changes: A 3-Year Progressive Study. Nutrients. 2020;12(9) doi: 10.3390/nu12092644.

[7] Duan W, Guo Z, Jiang H, Ware M, Li XJ, Mattson MP. Dietary restriction normalizes glucose metabolism and BDNF levels, slows disease progression, and increases survival in huntingtin mutant mice. Proc Natl Acad Sci U S A. 2003;100(5):2911-6 doi: 10.1073/pnas.0536856100.

[8] Ehrnhoefer DE, Martin DDO, Schmidt ME, Qiu X, Ladha S, Caron NS, et al. Preventing mutant huntingtin proteolysis and intermittent fasting promote autophagy in models of Huntington disease. Acta Neuropathol Commun. 2018;6(1):16 doi: 10.1186/s40478-018-0518-0.

[9] Wang HB, Loh DH, Whittaker DS, Cutler T, Howland D, Colwell CS. Time-Restricted Feeding Improves Circadian Dysfunction as well as Motor Symptoms in the Q175 Mouse Model of Huntington's Disease. eNeuro. 2018;5(1) doi: 10.1523/eneuro.0431-17.2017.

[10] Whittaker DS, Loh DH, Wang HB, Tahara Y, Kuljis D, Cutler T, et al. Circadian-based Treatment Strategy Effective in the BACHD Mouse Model of Huntington's Disease. J Biol Rhythms. 2018;33(5):535-54 doi: 10.1177/0748730418790401.

[11] Gabel K, Hoddy KK, Haggerty N, Song J, Kroeger CM, Trepanowski JF, et al. Effects of 8-hour time restricted feeding on body weight and metabolic disease risk factors in obese adults: A pilot study. Nutr Healthy Aging. 2018;4(4):345-53 doi: 10.3233/nha-170036.

[12] Martens CR, Rossman MJ, Mazzo MR, Jankowski LR, Nagy EE, Denman BA, et al. Short-term time-restricted feeding is safe and feasible in non-obese healthy midlife and older adults. Geroscience. 2020;42(2):667-86 doi: 10.1007/s11357-020-00156-6.

[13] Johri A, Calingasan NY, Hennessey TM, Sharma A, Yang L, Wille E, et al. Pharmacologic activation of mitochondrial biogenesis exerts widespread beneficial effects in a transgenic mouse model of Huntington's disease. Hum Mol Genet. 2012;21(5):1124-37 doi: 10.1093/hmg/ddr541.

[14] Pandey M, Rajamma U. Huntington's disease: the coming of age. J Genet. 2018;97(3):649-64 doi:

[15] Podvin S, Reardon HT, Yin K, Mosier C, Hook V. Multiple clinical features of Huntington's disease correlate with mutant HTT gene CAG repeat lengths and neurodegeneration. J Neurol. 2019;266(3):551-64 doi: 10.1007/s00415-018-8940-6.

[16] van der Burg JM, Bjorkqvist M, Brundin P. Beyond the brain: widespread pathology in Huntington's disease. Lancet Neurol. 2009;8(8):765-74 doi: 10.1016/S1474-4422(09)70178-4.

[17] Smarr B, Cutler T, Loh DH, Kudo T, Kuljis D, Kriegsfeld L, et al. Circadian dysfunction in the Q175 model of Huntington's disease: Network analysis. J Neurosci Res. 2019;97(12):1606-23 doi: 10.1002/jnr.24505.

[18] Gusella JF, MacDonald ME, Lee JM. Genetic modifiers of Huntington's disease. Mov Disord. 2014;29(11):1359-65 doi: 10.1002/mds.26001.

[19] Langbehn DR, Hayden MR, Paulsen JS, and the P-HDIotHSG. CAG-repeat length and the age of onset in Huntington disease (HD): a review and validation study of statistical approaches. Am J Med Genet B Neuropsychiatr Genet. 2010;153B(2):397-408 doi: 10.1002/ajmg.b.30992.

[20] Wexler NS, Lorimer J, Porter J, Gomez F, Moskowitz C, Shackell E, et al. Venezuelan kindreds reveal that genetic and environmental factors modulate Huntington's disease age of onset. Proc Natl Acad Sci U S A. 2004;101(10):3498-503 doi: 10.1073/pnas.0308679101.

[21] Trembath MK, Horton ZA, Tippett L, Hogg V, Collins VR, Churchyard A, et al. A retrospective study of the impact of lifestyle on age at onset of Huntington disease. Mov Disord. 2010;25(10):1444-50 doi: 10.1002/mds.23108.

[22] Consortium GMoHsDG-H. CAG Repeat Not Polyglutamine Length Determines Timing of Huntington's Disease Onset. Cell. 2019;178(4):887-900.e14 doi: 10.1016/j.cell.2019.06.036.

[23] Consortium GMoHsDG-H. Identification of Genetic Factors that Modify Clinical Onset of Huntington's Disease. Cell. 2015;162(3):516-26 doi: 10.1016/j.cell.2015.07.003.

[24] Phillips MCL, McManus EJ, Brinkhuis M, Romero-Ferrando B. Time-Restricted Ketogenic Diet in Huntington's Disease: A Case Study. Front Behav Neurosci. 2022;16:931636 doi: 10.3389/fnbeh.2022.931636.

[25] Ogilvie AC, Nopoulos PC, Schultz JL. Quantifying the Onset of Unintended Weight Loss in Huntington's Disease: A Retrospective Analysis of Enroll-HD. J Huntingtons Dis. 2021;10(4):485-92 doi: 10.3233/jhd-210488.

[26] Byrne LM, Rodrigues FB, Blennow K, Durr A, Leavitt BR, Roos RAC, et al. Neurofilament light protein in blood as a potential biomarker of neurodegeneration in Huntington's disease: a retrospective cohort analysis. Lancet Neurol. 2017;16(8):601-9 doi: 10.1016/s1474-4422(17)30124-2.

[27] Byrne LM, Rodrigues FB, Johnson EB, Wijeratne PA, De Vita E, Alexander DC, et al. Evaluation of mutant huntingtin and neurofilament proteins as potential markers in Huntington's disease. Sci Transl Med. 2018;10(458) doi: 10.1126/scitranslmed.aat7108.

[28] You H, Wu T, Du G, Huang Y, Zeng Y, Lin L, et al. Evaluation of Blood Glial Fibrillary Acidic Protein as a Potential Marker in Huntington's Disease. Front Neurol. 2021;12:779890 doi: 10.3389/fneur.2021.779890.

[29] Zhang S, Cheng Y, Shang H. The updated development of blood-based biomarkers for Huntington's disease. J Neurol. 2023;270(5):2483-503 doi: 10.1007/s00415-023-11572-x.

[30] Weiss A, Träger U, Wild EJ, Grueninger S, Farmer R, Landles C, et al. Mutant huntingtin fragmentation in immune cells tracks Huntington's disease progression. J Clin Invest. 2012;122(10):3731-6 doi: 10.1172/jci64565.

[31] Hensman Moss DJ, Robertson N, Farmer R, Scahill RI, Haider S, Tessari MA, et al. Quantification of huntingtin protein species in Huntington's disease patient leukocytes using optimised electrochemiluminescence immunoassays. PLoS One. 2017;12(12):e0189891 doi: 10.1371/journal.pone.0189891.

[32] Erlangga Z, Ghashang SK, Hamdan I, Melk A, Gutenbrunner C, Nugraha B. The effect of prolonged intermittent fasting on autophagy, inflammasome and senescence genes expressions: An exploratory study in healthy young males. Human Nutrition & Metabolism. 2023;32:200189 doi: <https://doi.org/10.1016/j.hnm.2023.200189>.

[33] Ganley IG, Lam du H, Wang J, Ding X, Chen S, Jiang X. ULK1.ATG13.FIP200 complex mediates mTOR signaling and is essential for autophagy. J Biol Chem. 2009;284(18):12297-305 doi: 10.1074/jbc.M900573200.

[34] Neilson LE, Quinn JF, Gray NE. Peripheral Blood NRF2 Expression as a Biomarker in Human Health and Disease. Antioxidants (Basel). 2020;10(1) doi: 10.3390/antiox10010028.

[35] Tucci P, Lattanzi R, Severini C, Saso L. Nrf2 Pathway in Huntington's Disease (HD): What Is Its Role? Int J Mol Sci. 2022;23(23) doi: 10.3390/ijms232315272.

[36] Madkour MI, A TE-S, Jahrami HA, Sherif NM, Hassan RE, Awadallah S, Faris MAE. Ramadan diurnal intermittent fasting modulates SOD2, TFAM, Nrf2, and sirtuins (SIRT1, SIRT3) gene expressions in subjects with overweight and obesity. Diabetes Res Clin Pract. 2019;155:107801 doi: 10.1016/j.diabres.2019.107801.

[37] Jin YN, Johnson GV. The interrelationship between mitochondrial dysfunction and transcriptional dysregulation in Huntington disease. J Bioenerg Biomembr. 2010;42(3):199-205 doi: 10.1007/s10863-010-9286-7.
