# Supplementary material for: Time-restricted eating in early-stage Huntington’s disease: A 12-week interventional clinical trial protocol
Source: PLoS One. 2025 Mar 25;20(3):e0319253. doi: 10.1371/journal.pone.0319253 (PMC11936236; doi:10.1371/journal.pone.0319253)
Supplement: File S3 — (DOCX) [file pone.0319253.s003.docx]

**Daily Survey**

Good evening! Welcome to your Nightly Survey. Please answer all of the following questions.

Subject number (Ex. TREHD_00x): ______________

Please enter today’s date and time: _____________

1. At approximately what time today did you first consume calories (food or beverage)?

_________

1. At approximately what time today did you stop consuming calories (food or beverage)?

_________

1. At approximately what time did you go to bed last night?

_________

1. At approximately what time did you wake up this morning?

_________

1. Approximately how long did it take to fall asleep last night?

_________

1. Approximately how much time did you spend napping today?

_________

**Weekly Survey**

Hello! Welcome to your Weekly Survey. Please answer all of the following questions.

Subject number (Ex. TREHD_00X): __________________

Please enter today’s date and time: ___________________

1. What was your body weight this week from any morning before eating? __________
2. How would you rate your overall sleep quality over the course of the last week?
   - Very bad
   - Fairly bad
   - Fairly good
   - Very good
3. In the past week, how would you rate your fatigue on average?
   - None
   - Mild
   - Moderate
   - Severe
   - Very severe
4. In the past week, how often have you been bothered by emotional problems such as feeling anxious, depressed, or irritable?
   - Never
   - Rarely
   - Sometimes
   - Often
   - Always
5. How many days in the last week did you exercise or take part in **vigorous** physical activities that made you sweat and breathe hard for at least 30 minutes (such as basketball, jogging, swimming laps, tennis, fast bicycling, strength training or similar)?
   - 0
   - 1
   - 2
   - 3
   - 4
   - 5
   - 6
   - 7
6. How many days in the last week did you exercise or take part in **moderate** physical activities that increased your breathing for a total of at least 30 minutes (such as brisk walking)? Do not include activities from question above.
   - 0
   - 1
   - 2
   - 3
   - 4
   - 5
   - 6
   - 7
7. How many days in the last week did you exercise to **strengthen or tone** your muscles (such as push-ups, sit-ups, or weightlifting)? This can include activities you counted in the above questions.
   - 0
   - 1
   - 2
   - 3
   - 4
   - 5
   - 6
   - 7
8. Thinking of all your physical activity added together, how much **total time** over the past week did you spend doing moderate to vigorous physical activities? (such as brisk walking, strength training, basketball, jogging, swimming laps, tennis, fast bicycling, or similar)

(In minutes) __________________

1. How likely are you to nod off or fall asleep in the following situations, in contrast to feeling just tired? This refers to your usual way of life in the past week.

   Even if you haven’t done some of these things recently, try to work out how they would have affected you. It is important that you answer each question as best you can.

   Use the following scale to choose the most appropriate statement for each situation.


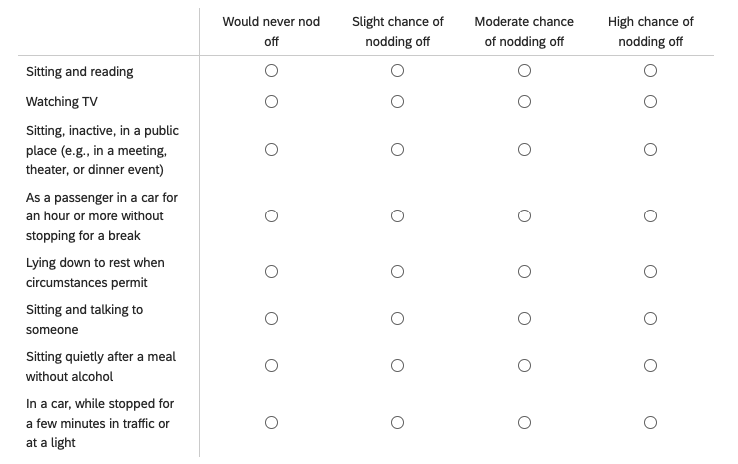


**In-lab Baseline Survey**

Time-Restricted Eating in Huntington’s Disease

In-laboratory Baseline Survey

***Your answers are completely confidential.***

***After your survey is recorded, this page with your name will be removed.***

***Only an ID Code will remain with your answers.***

***This survey will take 20-30 minutes.***

| *ID#: ___ ____* | | | Date: | _ _/_ _/__ __ |
| --- | --- | --- | --- | --- |
| Received: | ____________ (staff initials) | Time: | | _____:_____ am/pm |

OHSU IRB # 26970

Amie Hiller, Principal Investigator

This Page Intentionally Left Blank

**The following survey will ask you questions about your sleep, diet habits, physical activity, and demographics.**

**Your responses are very important to us; thank you for participating.**

**This first section is about your sleep habits, during the past month only. Your answers should indicate the most accurate reply for the majority of days and nights in the past month. Please answer all questions.**

**1.** During the past month, what time have you usually gone to bed at night?

BED TIME __ __: __ __ AM/PM (circle one).

**2.** During the past month, how long (in minutes) has it usually taken you to fall asleep each night?

NUMBER OF MINUTES ___________

**3.** During the past month, what time have you usually gotten up in the morning?

GETTING UP TIME __ __: __ __ AM/PM (circle one).

**4.** During the past month, how many hours of actual sleep did you get at night? (This may be different than the number of hours you spent in bed.)

HOURS OF SLEEP PER NIGHT _________

*For each of the remaining questions, check the one best response. Please answer all questions.*

**5.** During the past month, how often have you had trouble sleeping because you…

1. Cannot get to sleep within 30 minutes

- Not during the past month
- Less than once a week
- 1-2 times a week
- 3 or more times a week

1. Wake up in the middle of the night or early morning

- Not during the past month
- Less than once a week
- 1-2 times a week
- 3 or more times a week

1. Have to get up to use the bathroom

- Not during the past month
- Less than once a week
- 1-2 times a week
- 3 or more times a week

1. Cannot breathe comfortably

- Not during the past month
- Less than once a week
- 1-2 times a week
- 3 or more times a week

1. Cough or snore loudly

- Not during the past month
- Less than once a week
- 1-2 times a week
- 3 or more times a week

1. Feel too cold

- Not during the past month
- Less than once a week
- 1-2 times a week
- 3 or more times a week

1. Feel too hot

- Not during the past month
- Less than once a week
- 1-2 times a week
- 3 or more times a week

1. Had bad dreams

- Not during the past month
- Less than once a week
- 1-2 times a week
- 3 or more times a week

1. Have pain

- Not during the past month
- Less than once a week
- 1-2 times a week
- 3 or more times a week

1. Other reason(s), please describe: ________________________________________________________________________________________________________________________________________________

How often during the past month have you had trouble sleeping because of this?

- Not during the past month
- Less than once a week
- 1-2 times a week
- 3 or more times a week

**6.** During the past month, how would you rate your sleep quality overall?

- Very good
- Fairly good
- Fairly bad
- Very bad

**7.** During the past month, how often have you taken medicine to help you sleep (prescribed or “over the counter”)?

- Not during the past month
- Less than once a week
- 1-2 times a week
- 3 or more times a week

**8.** During the past month, how often have you had trouble staying awake while driving, eating meals, or engaging in social activity?

- Not during the past month
- Less than once a week
- 1-2 times a week
- 3 or more times a week

**9.** During the past month, how much of a problem has it been for you to keep up enough enthusiasm to get things done?

- No problem at all
- Only a very slight problem
- Somewhat of a problem
- A very big problem

**10.** Do you have a bed partner of roommate?

- No bed partner or roommate
- Partner/roommate in other room
- Partner in same room, but not same bed
- Partner in same bed

If you have a roommate or bed partner, ask him/her how often in the past month you have had…

1. Loud snoring

- Not during the past month
- Less than once a week
- 1-2 times a week
- 3 or more times a week

1. Long pauses between breaths while asleep

- Not during the past month
- Less than once a week
- 1-2 times a week
- 3 or more times a week

1. Legs twitching or jerking while you sleep

- Not during the past month
- Less than once a week
- 1-2 times a week
- 3 or more times a week

1. Episodes of disorientation or confusion during sleep

- Not during the past month
- Less than once a week
- 1-2 times a week
- 3 or more times a week

1. Other restlessness while you sleep: please describe: ________________________________________________________________________________________________________________________________________________

- Not during the past month
- Less than once a week
- 1-2 times a week
- 3 or more times a week

**This section is about your eating habits, answer each question to the best of your ability.**

1. Think about your eating habits over the **past 3 months**. About how often did you eat or drink each of the following foods? Remember breakfast, lunch, dinner, snacks, and eating out. Mark only one bubble for each food.

| **Type of Food** | **Never** | **Less than once** | **1-3 times** | **1-2 times per week** | **3-4 times per week** | **5-6 times per week** | **Once a day** | **2 times per day** | **3-4 times per day** | **5 or more times per day** |
| --- | --- | --- | --- | --- | --- | --- | --- | --- | --- | --- |
| **Sugary snacks**  (*such as cake, sweet rolls, pastries, donuts, cookies, brownies, pie, and candy)* | 🞎 | 🞎 | 🞎 | 🞎 | 🞎 | 🞎 | 🞎 | 🞎 | 🞎 | 🞎 |
| **Drinks with added sugar**  *(such as regular soda, sport drinks, coffee, iced tea, lemonade, and fruit punch)* | 🞎 | 🞎 | 🞎 | 🞎 | 🞎 | 🞎 | 🞎 | 🞎 | 🞎 | 🞎 |
| **Fast Food**  *(such as McDonalds, Burger King, Burgerville, Taco Bell, and Wendys)* | 🞎 | 🞎 | 🞎 | 🞎 | 🞎 | 🞎 | 🞎 | 🞎 | 🞎 | 🞎 |
| **Meals from home** *(such as those you bring to work for breakfast, lunch or dinner)* | 🞎 | 🞎 | 🞎 | 🞎 | 🞎 | 🞎 | 🞎 | 🞎 | 🞎 | 🞎 |
| **Meats**  *(such as hamburgers, beef steaks, pork, or hot dogs)* | 🞎 | 🞎 | 🞎 | 🞎 | 🞎 | 🞎 | 🞎 | 🞎 | 🞎 | 🞎 |

**Thank you for your answers so far!**

| **INSTRUCTIONS:** Think about what you usually ate in the **last 3 months**. Please think about all the fruits and vegetables that you ate in the last 3 months. Include those that were: a) raw and cooked; b) eaten as snacks and at meals; c) eaten at home and away from home (restaurants, friends, take-out); and d) eaten alone and mixed with other foods. Report how many times per month, week or day you ate each food and, if you ate it, how much you usually had. If you mark “Never” for a question, follow the “Go to” instruction. **Choose the best answer** for each question. **Mark only one** response for each question. | | | | | | | | | | | | | | | | |
| --- | --- | --- | --- | --- | --- | --- | --- | --- | --- | --- | --- | --- | --- | --- | --- | --- |
| 1. **Over the last 3 months**, how many times per month, week, or day did you drink **100% juice** such as orange, apple, grape, or grapefruit juice? Do not count fruit drinks like Kool-Aid, lemonade, Hi-C, cranberry juice drink, or Twister. Include juice you drank at all mealtimes and between meals. | | | | | | | | | | | | | | |  |  |
| Ο  Never  (Go to Q2) | Ο  1-3 times per **month** | Ο  1-2 times per **week** | | | Ο  3-4 times per **week** | Ο  5-6 times per **week** | | Ο  1 time per **day** | Ο  2 times per **day** | Ο  3 times per **day** | | | Ο  4 times per **day** | Ο  5 or more times/**day** | |  |
| **1a**. Each time you drank **100% juice**, how much did you usually drink? | | | | | | | | | | | | | | | |  |
| Ο  Less than ¾ cup  (less than 6 ounces) | | | | Ο  ¾ to 1¼ cup  (6 to 10 ounces) | | | | Ο  1¼ to 2 cups  (10 to 16 ounces) | | | | Ο  More than 2 cups  (More than 16 ounces) | | | |  |
| 1. **Over the last 3 months**, how many times per month, week, or day did you eat **fruit**? Count any kind of fruit – fresh, canned, and frozen. Do not count juices. Include fruit you ate at all mealtimes and snacks. | | | | | | | | | | | | | | | |  |
| Ο  Never  (Go to Q3) | Ο  1-3 times per **month** | Ο  1-2 times per **week** | | | Ο  3-4 times per **week** | Ο  5-6 times per **week** | | Ο  1 time per **day** | Ο  2 times per **day** | Ο  3 times per **day** | | | Ο  4 times per **day** | Ο  5 or more times/**day** | |  |
| **2a**. Each time you ate **fruit**, how much did you usually eat? | | | | | | | | | | | | | | | |  |
| Ο Less than 1 medium fruit  **OR**  Ο  Less than ½ cup | | | Ο 1 medium fruit  **OR**  Ο  About ½ cup | | | | Ο 2 medium fruits  **OR**  Ο  About 1 cup | | | | Ο More than 2 medium fruits  **OR**  Ο  More than 1 cup | | | | |  |

| **3.** **Over the last 3 months**, how often did you eat **lettuce salad** (with or without other vegetables)? | | | | | | | | | |
| --- | --- | --- | --- | --- | --- | --- | --- | --- | --- |
| Ο  Never  (Go to Q4) | Ο  1-3 times per **month** | Ο  1-2 times per **week** | Ο  3-4 times per **week** | Ο  5-6 times per **week** | Ο  1 time per **day** | Ο  2 times per **day** | Ο  3 times per **day** | Ο  4 times per **day** | Ο  5 or more times/**day** |

| **3a**. Each time you ate **lettuce salad**, how much did you usually eat? | | | | | | | | | | | |
| --- | --- | --- | --- | --- | --- | --- | --- | --- | --- | --- | --- |
| Ο  About ½ cup | | | Ο  About 1 cup | | | Ο  About 2 cups | | | Ο  More than 2 cups | | |
| **4.** **Over the last 3 months**, how often did you eat **French fries** or **fried potatoes**? | | | | | | | | | | | |
| Ο  Never  (Go to Q5) | Ο  1-3 times per **month** | Ο  1-2 times per **week** | | Ο  3-4 times per **week** | Ο  5-6 times per **week** | Ο  1 time per **day** | Ο  2 times per **day** | Ο  3 times per **day** | | Ο  4 times per **day** | Ο  5 or more times/**day** |
| **4a.** Each time you ate **French fries** or **fried potatoes**, how much did you usually eat? | | | | | | | | | | | |
| Ο  Small Order or Less  (About 1 cup or less) | | | Ο  Medium Order  (About 1½ cups) | | | Ο  Large order  (About 2 cups) | | | Ο  Supersize order or more  (About 3 cups or more) | | |
| **5.** **Over the last 3 months**, how often did you eat other white potatoes? Count **baked, boiled**, and **mashed potatoes**, **potato salad**, and **white potatoes that were not fried**. | | | | | | | | | | | |
| Ο  Never  (Go to Q6) | Ο  1-3 times per **month** | Ο  1-2 times per **week** | | Ο  3-4 times per **week** | Ο  5-6 times per **week** | Ο  1 time per **day** | Ο  2 times per **day** | Ο  3 times per **day** | | Ο  4 times per **day** | Ο  5 or more times/**day** |
| **5a.** Each time you ate **these potatoes**, how much did you usually eat? | | | | | | | | | | | |
| Ο  1 small potato or less  (½ cup or less) | | | Ο  1 medium potato  (½ to 1 cup) | | | Ο  1 large potato  (1 to 1½ cups) | | | Ο  2 medium potatoes or more  (1½ cups or more) | | |
| **6.** **Over the last 3 months**, how often did you eat **cooked dried beans**? Count **baked beans, bean soup, refried beans, pork and beans**, and **other bean dishes**. | | | | | | | | | | | |
| Ο  Never  (Go to Q7) | Ο  1-3 times per **month** | Ο  1-2 times per **week** | | Ο  3-4 times per **week** | Ο  5-6 times per **week** | Ο  1 time per **day** | Ο  2 times per **day** | Ο  3 times per **day** | | Ο  4 times per **day** | Ο  5 or more times/**day** |
| **6a**. Each time you ate **these beans**, how much did you usually eat? | | | | | | | | | | | |
| Ο  Less than ½ cup | | | Ο  ½ to 1 cup | | | Ο  1 to 1½ cups | | | Ο  More than 1½ cups | | |

| **7.** **Over the last 3 months**, how often did you eat **other vegetables**? | | | | | | | | | | | | | | | | | | | | | | |
| --- | --- | --- | --- | --- | --- | --- | --- | --- | --- | --- | --- | --- | --- | --- | --- | --- | --- | --- | --- | --- | --- | --- |
| DO NOT COUNT:   - Lettuce salads - White potatoes - Cooked dried beans - Vegetables in mixtures, such as sandwiches, omelets, Mexican dishes, stews, stir-fries, soups, etc. - Rice   COUNT:   - All other vegetables – raw, cooked, canned, and frozen | | | | | | | | | | | | | | | | | | | | | | |
| Ο  Never  (Go to Q8) | Ο  1-3 times per **month** | | Ο  1-2 times per **week** | | | | Ο  3-4 times per **week** | | Ο  5-6 times per **week** | | Ο  1 time per **day** | | Ο  2 times per **day** | | Ο  3 times per **day** | | | | Ο  4 times per **day** | | Ο  5 or more times/**day** | |
| **7a.** Each time you ate **other vegetables**, how much did you usually eat? | | | | | | | | | | | | | | | | | | | | | | |
| Ο  Less than ½ cup | | | | | Ο  ½ to 1 cup | | | | | | Ο  1 to 1½ cups | | | | | | Ο  More than 1½ cups | | | | | |
| **8.** **Over the last 3 months**, how often did you eat **tomato sauce**? Include tomato sauce on pasta or   macaroni, rice, pizza and other dishes. | | | | | | | | | | | | | | | | | | | | | | |
| Ο  Never  (Go to Q9) | Ο  1-3 times per **month** | | Ο  1-2 times per **week** | | | | Ο  3-4 times per **week** | | Ο  5-6 times per **week** | | Ο  1 time per **day** | | Ο  2 times per **day** | | Ο  3 times per **day** | | | | Ο  4 times per **day** | | Ο  5 or more times/**day** | |
| **8a.** Each time you ate **tomato sauce**, how much did you usually eat? | | | | | | | | | | | | | | | | | | | | | | |
| Ο  About ¼ cup | | | | | Ο  About ½ cup | | | | | | Ο  About 1 cup | | | | | | Ο  More than 1 cup | | | | | |
| **9.** **Over the last 3 months**, how often did you eat **vegetable soups**? Include tomato soup, gazpacho,   beef with vegetable soup, minestrone soup, and other soups made with vegetables. | | | | | | | | | | | | | | | | | | | | | | |
| Ο  Never  (Go to Q10) | Ο  1-3 times per **month** | | Ο  1-2 times per **week** | | | | Ο  3-4 times per **week** | | Ο  5-6 times per **week** | | Ο  1 time per **day** | | Ο  2 times per **day** | | Ο  3 times per **day** | | | | Ο  4 times per **day** | | Ο  5 or more times/**day** | |
| **9a.** Each time you ate **vegetable soup**, how much did you usually eat? | | | | | | | | | | | | | | | | | | | | | | |
| Ο  Less than 1 cup | | | | | | Ο  1 to 2 cups | | | | | | Ο  2 to 3 cups | | | | | | Ο  More than 3 cups | | | | |
| **10.** **Over the last 3 months**, how often did you eat **mixtures that included vegetables**? Count such foods as sandwiches, casseroles, stews, stir-fries, omelets, and tacos. | | | | | | | | | | | | | | | | | | | | | | |
| Ο  Never | | Ο  1-3 times per **month** | | Ο  1-2 times per **week** | | | | Ο  3-4 times per **week** | | Ο  5-6 times per **week** | | Ο  1 time per **day** | | Ο  2 times per **day** | | Ο  3 times per **day** | | | | Ο  4 times per **day** | | Ο  5 or more times/**day** |

**11.** **Over the last 3 months**, at what time of day did you typically first consume any food/calories (this includes liquids with calories)? __ __: __ __ AM/PM (circle one).

**12.** **Over the last 3 months**, at what time of day did you typically consume your last food/calories (this includes liquids with calories)? __ __: __ __ AM/PM (circle one).

**12a.** For how long have you maintained the schedule listed above?

| Ο  < 1 month | Ο  1-3 months | Ο  4-6 months | Ο  7-12 months | Ο  1-5 years | Ο  5-10 years | Ο  > 10 years |
| --- | --- | --- | --- | --- | --- | --- |

**13. Over the last 3 months**, have you attempted to follow any specific diet?

Other (please specify): ________________________________________________________________

| Ο  No specific diet (skip to Q 14) | Ο  Low carb diet | Ο  Low-calorie diet | Ο  Low-fat  diet | Ο  Keto diet | Ο  DASH diet | Ο  Mediterran-ean diet | Ο  Vegetarian diet | Ο  Gluten free diet | Ο  Vegan diet |
| --- | --- | --- | --- | --- | --- | --- | --- | --- | --- |

**13a.** For how long have you attempted to maintain this diet?

| Ο  < 1 month | Ο  1-3 months | Ο  4-6 months | Ο  7-12 months | Ο  1-5 years | Ο  5-10 years | Ο  > 10 years |
| --- | --- | --- | --- | --- | --- | --- |

**13b.** How would you rate your adherence to this diet?

| Ο  Poor  < 25% of meals | Ο  Fair  < 50% of meals | Ο  Good  > 50% of meals | Ο  Very Good  > 75% of meals | Ο  Excellent  > 90% of meals |
| --- | --- | --- | --- | --- |

**14. Over the last 3 months**, have you practiced any form of dietary fasting protocol (completely and regularly avoiding caloric intake for a certain length of time)?

- Yes
- No (Go to Q15)

**14a.** What was the pattern of fasting?

Other (please specify): _____________________________________________________________

| Ο  Daily  12 hours fasting  12 hours eating | Ο  Daily  16 hours fasting  8 hours eating | Ο  Daily  18 hours fasting  6 hours eating | Ο  Alternate day fasting | Ο  Fasting two days per week  (5:2 diet) |
| --- | --- | --- | --- | --- |

**14b.** For how long did you, or have you, maintained the fasting protocol?

| Ο  < 1 month | Ο  1-3 months | Ο  4-6 months | Ο  7-12 months | Ο  1-5 years | Ο  5-10 years | Ο  > 10 years |
| --- | --- | --- | --- | --- | --- | --- |

**14c.** Why did you previously, or do you currently, practice a fasting protocol?

| Ο  To lose weight | Ο  To be healthy | Ο  Religious reasons | Ο  Other (please specify): |
| --- | --- | --- | --- |

**15.** If you previously practiced a fasting protocol (more than six months ago), at approximately what age did this start and stop? _____ to _____.

**This section is about your health and physical activity habits, answer each question to the best of your ability.**

1. Please respond to each item by marking one box per row:

|  | **Poor** | **Fair** | **Good** | **Very Good** | **Excellent** |
| --- | --- | --- | --- | --- | --- |
| In general, would you say that your health is: |  |  |  |  |  |
| In general, would you say that your quality of life is: |  |  |  |  |  |
| In general, how would you rate your physical health? |  |  |  |  |  |
| In general, how would you rate your mental health, including your mood and your ability to think? |  |  |  |  |  |
| In general, how would you rate your satisfaction with your social activities and relationships? |  |  |  |  |  |

1. To what extent are you able to carry out your everyday physical activities such as walking,
   climbing stairs, carrying groceries, or moving a chair?

| - Not at all able | - A little able | - Moderately able | - Mostly able | - Completely able |
| --- | --- | --- | --- | --- |

1. In the past 3 months, how would you rate your pain on average? (Circle your answer)

**No pain** 1 2 3 4 5 6 7 8 9 10 **Worst pain imaginable**

1. In the past 3 months, how would you rate your fatigue on average?

| - None | - Mild | - Moderate | - Severe | - Very Severe |
| --- | --- | --- | --- | --- |

1. In the past 3 months, in general, please rate how well you carry out your usual social activities and roles. (This includes activities at home, at work and in your community, and responsibilities as a parent, child, spouse, employee, friend, etc.)

| - Poor | - Fair | - Good | - Very Good | - Excellent |
| --- | --- | --- | --- | --- |

1. In the past 3 months, how often have you been bothered by emotional problems such as feeling anxious, depressed or irritable?

| - Never | - Rarely | - Sometimes | - Often | - Always |
| --- | --- | --- | --- | --- |

1. In the past, did you smoke tobacco?

- Yes, daily
- Yes, less than daily
- No, not at all

1. Think about your use of the following products over the **last 3 months**. About how often did you consume each of the following? Mark only one frequency selection on each row.

|  | **Never** | **Less than once** | **1-3 times** | **1-2 times per week** | **3-4 times per week** | **5-6 times per week** | **Once per day** | **2 times per day** | **3-4 times per day** | **5+ times per day** |
| --- | --- | --- | --- | --- | --- | --- | --- | --- | --- | --- |
| Coffee | 🞎 | 🞎 | 🞎 | 🞎 | 🞎 | 🞎 | 🞎 | 🞎 | 🞎 | 🞎 |
| Tea | 🞎 | 🞎 | 🞎 | 🞎 | 🞎 | 🞎 | 🞎 | 🞎 | 🞎 | 🞎 |
| Caffeinated soft drinks, energy drinks  (i.e., Coke, Redbull) | 🞎 | 🞎 | 🞎 | 🞎 | 🞎 | 🞎 | 🞎 | 🞎 | 🞎 | 🞎 |
| Alcohol | 🞎 | 🞎 | 🞎 | 🞎 | 🞎 | 🞎 | 🞎 | 🞎 | 🞎 | 🞎 |
| Aspirin | 🞎 | 🞎 | 🞎 | 🞎 | 🞎 | 🞎 | 🞎 | 🞎 | 🞎 | 🞎 |
| Over the counter pain relievers | 🞎 | 🞎 | 🞎 | 🞎 | 🞎 | 🞎 | 🞎 | 🞎 | 🞎 | 🞎 |
| Cold medications | 🞎 | 🞎 | 🞎 | 🞎 | 🞎 | 🞎 | 🞎 | 🞎 | 🞎 | 🞎 |
| Allergy medicine/ antihistamines | 🞎 | 🞎 | 🞎 | 🞎 | 🞎 | 🞎 | 🞎 | 🞎 | 🞎 | 🞎 |
| Sleeping pills/tranquilizers | 🞎 | 🞎 | 🞎 | 🞎 | 🞎 | 🞎 | 🞎 | 🞎 | 🞎 | 🞎 |

1. Please answer the next four questions about physical activity as they have applied to you over the **past 3 months:**

**9a.** How many days per week did you exercise or take part in **vigorous** physical activities that made you sweat and breathe hard for at least 30 minutes (such as basketball, jogging, swimming laps, tennis, fast bicycling, strength training, or similar)?

Please circle one: 0 1 2 3 4 5 6 7

**9b.** How many days per week did you exercise or take part in **moderate** physical activities that increased your breathing for a total of at least 30 minutes (such as brisk walking)? *Do not include activities from question above.*

Please circle one: 0 1 2 3 4 5 6 7

**9c.** How many days per week did you exercise to **strengthen or tone** your muscles (such as push-ups, sit-ups, or weight lifting)? *This can include activities you counted in the above questions.*

Please circle one: 0 1 2 3 4 5 6 7

**9d.** In a typical week, how many days do you take part in any physical activity long enough to work up a sweat? *This can include activities counted in questions above.*

Please circle one: 0 1 2 3 4 5 6 7

1. Please describe all exercise or athletic activities in which you participate regularly:

| Write answer here: |
| --- |

1. **How would you describe your typical day in the last 3 months?**

__________ % Sitting (including driving)

__________ % Standing

__________ % Walking

__________ % Heavy labor or physically demanding tasks

=100% total

**This is the last section.**

**You are almost done!**

**1.** What is your gender?

- Male
- Female
- Other/non-binary
- Prefer not to say

**2.** What is your age in years? __________Years

**3.** What is your highest level of education?

- Never attended school, or only kindergarten
- Grades 1-8 (Elementary)
- Grades 9-11 (Some high school)
- Grade 12 or GED (High school graduate)
- College 1 year to 3 years (Some college or technical school)
- College 4 years or more (College graduate)
- Graduate school post college (Masters or doctoral)
- Professional degree (eg., J.D., M.D.)

**4.** What is your ethnic background?

- Hispanic/Latino
- Not Hispanic/Latino

**5.** What’s your racial background?

- American Indian/Alaska Native
- Asian
- Black/African American
- Native Hawaiian/Pacific Islander
- White/Caucasian
- More than one race
- Other (Specify)__________

**6.** What is your current relationship status?

- Married
- Divorced or separated in past year
- Widowed
- Living with significant other
- Single

**7.** Please note each major life event that you have experienced in the last 3 months:

| **Life Event** | **Yes** | **No** |
| --- | --- | --- |
| Death of spouse |  |  |
| Divorce |  |  |
| Marital separation from mate |  |  |
| Detention in jail or other institution |  |  |
| Death of a close family member |  |  |
| Major personal injury or illness |  |  |
| Marriage |  |  |
| Being fired at work |  |  |
| Marital reconciliation with mate |  |  |
| Retirement from work |  |  |
| Major change in health or behavior of a family member |  |  |
| Pregnancy |  |  |
| Sexual difficulties |  |  |
| Gaining a new family member (i.e. birth, adoption, older adult moving in, etc.) |  |  |
| Major business readjustment |  |  |
| Major change in financial state (i.e. a lot worse or better off than usual) |  |  |
| Death of a close friend |  |  |
| Changing to a different line of work |  |  |
| Major change in the number of arguments w/spouse (i.e. either a lot more or a lot less than usual regarding child rearing, personal habits, etc.) |  |  |
| Taking on a mortgage |  |  |
| Foreclosure on a mortgage or loan |  |  |
| Major change in responsibilities at work (i.e. promotion, demotion, etc.) |  |  |
| Son or daughter leaving home (marriage, attending college, joined military) |  |  |
| In-law troubles |  |  |
| Outstanding personal achievement |  |  |
| Spouse beginning or ceasing work outside the home |  |  |
| Beginning or ceasing formal schooling |  |  |
| Major change in living condition (new home, remodeling, deterioration of neighborhood or home etc.) |  |  |
| Revision of personal habits (dress manners, associations, quitting smoking) |  |  |
| Troubles with the boss |  |  |
| Major changes in working hours or conditions |  |  |
| Changes in residence |  |  |
| Changing to a new school |  |  |
| Major change in usual type and/or amount of recreation |  |  |
| Major change in church activity (i.e. a lot more or less than usual) |  |  |
| Major change in social activities (clubs, movies, visiting, etc.) |  |  |
| Taking on a loan (car, TV, freezer, etc.) |  |  |
| Major change in sleeping habits (a lot more or less than usual) |  |  |
| Major change in number of family get-togethers (a lot more or less than normal) |  |  |
| Major change in eating habits (a lot more or less intake, or very different meal hours or surroundings) |  |  |
| Vacation |  |  |
| Major holidays |  |  |
| Minor violations of the law (traffic tickets, jaywalking, disturbing the peace, etc.) |  |  |

**Thank you for taking this survey!**

**Please alert the research staff when you are done.**

**In-lab Follow-up Survey**

Time-Restricted Eating in Huntington’s Disease

In-laboratory Follow-up Survey

***Your answers are completely confidential.***

***After your survey is recorded, this page with your name will be removed.***

***Only an ID Code will remain with your answers.***

***This survey will take 20-30 minutes.***

| *ID#: ___ ____* | | | Date: | _ _/_ _/__ __ |
| --- | --- | --- | --- | --- |
| Received: | ____________ (staff initials) | Time: | | _____:_____ am/pm |

OHSU IRB # 26970

Amie Hiller, Principal Investigator

This Page Intentionally Left Blank

**This first section is about your sleep habits, during the past month only. Your answers should indicate the most accurate reply for the majority of days and nights in the past month. Please answer all questions.**

**1.** During the past month, what time have you usually gone to bed at night?

BED TIME __ __: __ __ AM/PM (circle one).

**2.** During the past month, how long (in minutes) has it usually taken you to fall asleep each night?

NUMBER OF MINUTES ___________

**3.** During the past month, what time have you usually gotten up in the morning?

GETTING UP TIME __ __: __ __ AM/PM (circle one).

**4.** During the past month, how many hours of actual sleep did you get at night? (This may be different than the number of hours you spent in bed.)

HOURS OF SLEEP PER NIGHT _________

*For each of the remaining questions, check the one best response. Please answer all questions.*

**5.** During the past month, how often have you had trouble sleeping because you…

1. Cannot get to sleep within 30 minutes

- Not during the past month
- Less than once a week
- 1-2 times a week
- 3 or more times a week

1. Wake up in the middle of the night or early morning

- Not during the past month
- Less than once a week
- 1-2 times a week
- 3 or more times a week

1. Have to get up to use the bathroom

- Not during the past month
- Less than once a week
- 1-2 times a week
- 3 or more times a week

1. Cannot breathe comfortably

- Not during the past month
- Less than once a week
- 1-2 times a week
- 3 or more times a week

1. Cough or snore loudly

- Not during the past month
- Less than once a week
- 1-2 times a week
- 3 or more times a week

1. Feel too cold

- Not during the past month
- Less than once a week
- 1-2 times a week
- 3 or more times a week

1. Feel too hot

- Not during the past month
- Less than once a week
- 1-2 times a week
- 3 or more times a week

1. Had bad dreams

- Not during the past month
- Less than once a week
- 1-2 times a week
- 3 or more times a week

1. Have pain

- Not during the past month
- Less than once a week
- 1-2 times a week
- 3 or more times a week

1. Other reason(s), please describe: ________________________________________________________________________________________________________________________________________________

How often during the past month have you had trouble sleeping because of this?

- Not during the past month
- Less than once a week
- 1-2 times a week
- 3 or more times a week

**6.** During the past month, how would you rate your sleep quality overall?

- Very good
- Fairly good
- Fairly bad
- Very bad

**7.** During the past month, how often have you taken medicine to help you sleep (prescribed or “over the counter”)?

- Not during the past month
- Less than once a week
- 1-2 times a week
- 3 or more times a week

**8.** During the past month, how often have you had trouble staying awake while driving, eating meals, or engaging in social activity?

- Not during the past month
- Less than once a week
- 1-2 times a week
- 3 or more times a week

**9.** During the past month, how much of a problem has it been for you to keep up enough enthusiasm to get things done?

- No problem at all
- Only a very slight problem
- Somewhat of a problem
- A very big problem

**10.** Do you have a bed partner of roommate?

- No bed partner or roommate
- Partner/roommate in other room
- Partner in same room, but not same bed
- Partner in same bed

If you have a roommate or bed partner, ask him/her how often in the past month you have had…

1. Loud snoring

- Not during the past month
- Less than once a week
- 1-2 times a week
- 3 or more times a week

1. Long pauses between breaths while asleep

- Not during the past month
- Less than once a week
- 1-2 times a week
- 3 or more times a week

1. Legs twitching or jerking while you sleep

- Not during the past month
- Less than once a week
- 1-2 times a week
- 3 or more times a week

1. Episodes of disorientation or confusion during sleep

- Not during the past month
- Less than once a week
- 1-2 times a week
- 3 or more times a week

1. Other restlessness while you sleep: please describe: ________________________________________________________________________________________________________________________________________________

- Not during the past month
- Less than once a week
- 1-2 times a week
- 3 or more times a week

**This section is about your eating habits, answer each question to the best of your ability.**

1. Think about your eating habits over the **past 3 months**. About how often did you eat or drink each of the following foods? Remember breakfast, lunch, dinner, snacks, and eating out. Mark only one bubble for each food.

| **Type of Food** | **Never** | **Less than once** | **1-3 times** | **1-2 times per week** | **3-4 times per week** | **5-6 times per week** | **Once a day** | **2 times per day** | **3-4 times per day** | **5 or more times per day** |
| --- | --- | --- | --- | --- | --- | --- | --- | --- | --- | --- |
| **Sugary snacks**  (*such as cake, sweet rolls, pastries, donuts, cookies, brownies, pie, and candy)* | 🞎 | 🞎 | 🞎 | 🞎 | 🞎 | 🞎 | 🞎 | 🞎 | 🞎 | 🞎 |
| **Drinks with added sugar**  *(such as regular soda, sport drinks, coffee, iced tea, lemonade, and fruit punch)* | 🞎 | 🞎 | 🞎 | 🞎 | 🞎 | 🞎 | 🞎 | 🞎 | 🞎 | 🞎 |
| **Fast Food**  *(such as McDonalds, Burger King, Burgerville, Taco Bell, and Wendys)* | 🞎 | 🞎 | 🞎 | 🞎 | 🞎 | 🞎 | 🞎 | 🞎 | 🞎 | 🞎 |
| **Meals from home** *(such as those you bring to work for breakfast, lunch or dinner)* | 🞎 | 🞎 | 🞎 | 🞎 | 🞎 | 🞎 | 🞎 | 🞎 | 🞎 | 🞎 |
| **Meats**  *(such as hamburgers, beef steaks, pork, or hot dogs)* | 🞎 | 🞎 | 🞎 | 🞎 | 🞎 | 🞎 | 🞎 | 🞎 | 🞎 | 🞎 |

**Thank you for your answers so far!**

| **INSTRUCTIONS:** Think about what you usually ate in the **last 3 months**. Please think about all the fruits and vegetables that you ate last month. Include those that were: a) raw and cooked; b) eaten as snacks and at meals; c) eaten at home and away from home (restaurants, friends, take-out); and d) eaten alone and mixed with other foods. Report how many times per month, week or day you ate each food and, if you ate it, how much you usually had. If you mark “Never” for a question, follow the “Go to” instruction. **Choose the best answer** for each question. **Mark only one** response for each question. | | | | | | | | | | | | | | | | |
| --- | --- | --- | --- | --- | --- | --- | --- | --- | --- | --- | --- | --- | --- | --- | --- | --- |
| 1. **Over the last 3 months**, how many times per month, week, or day did you drink **100% juice** such as orange, apple, grape, or grapefruit juice? Do not count fruit drinks like Kool-Aid, lemonade, Hi-C, cranberry juice drink, or Twister. Include juice you drank at all mealtimes and between meals. | | | | | | | | | | | | | | |  |  |
| Ο  Never  (Go to Q2) | Ο  1-3 times per **month** | Ο  1-2 times per **week** | | | Ο  3-4 times per **week** | Ο  5-6 times per **week** | | Ο  1 time per **day** | Ο  2 times per **day** | Ο  3 times per **day** | | | Ο  4 times per **day** | Ο  5 or more times/**day** | |  |
| **1a**. Each time you drank **100% juice**, how much did you usually drink? | | | | | | | | | | | | | | | |  |
| Ο  Less than ¾ cup  (less than 6 ounces) | | | | Ο  ¾ to 1¼ cup  (6 to 10 ounces) | | | | Ο  1¼ to 2 cups  (10 to 16 ounces) | | | | Ο  More than 2 cups  (More than 16 ounces) | | | |  |
| 1. **Over the last 3 months**, how many times per month, week, or day did you eat **fruit**? Count any kind of fruit – fresh, canned, and frozen. Do not count juices. Include fruit you ate at all mealtimes and snacks. | | | | | | | | | | | | | | | |  |
| Ο  Never  (Go to Q3) | Ο  1-3 times per **month** | Ο  1-2 times per **week** | | | Ο  3-4 times per **week** | Ο  5-6 times per **week** | | Ο  1 time per **day** | Ο  2 times per **day** | Ο  3 times per **day** | | | Ο  4 times per **day** | Ο  5 or more times/**day** | |  |
| **2a**. Each time you ate **fruit**, how much did you usually eat? | | | | | | | | | | | | | | | |  |
| Ο Less than 1 medium fruit  **OR**  Ο  Less than ½ cup | | | Ο 1 medium fruit  **OR**  Ο  About ½ cup | | | | Ο 2 medium fruits  **OR**  Ο  About 1 cup | | | | Ο More than 2 medium fruits  **OR**  Ο  More than 1 cup | | | | |  |

| **3.** **Over the last 3 months**, how often did you eat **lettuce salad** (with or without other vegetables)? | | | | | | | | | |
| --- | --- | --- | --- | --- | --- | --- | --- | --- | --- |
| Ο  Never  (Go to Q4) | Ο  1-3 times per **month** | Ο  1-2 times per **week** | Ο  3-4 times per **week** | Ο  5-6 times per **week** | Ο  1 time per **day** | Ο  2 times per **day** | Ο  3 times per **day** | Ο  4 times per **day** | Ο  5 or more times/**day** |

| **3a**. Each time you ate **lettuce salad**, how much did you usually eat? | | | | | | | | | | | |
| --- | --- | --- | --- | --- | --- | --- | --- | --- | --- | --- | --- |
| Ο  About ½ cup | | | Ο  About 1 cup | | | Ο  About 2 cups | | | Ο  More than 2 cups | | |
| **4.** **Over the last 3 months**, how often did you eat **French fries** or **fried potatoes**? | | | | | | | | | | | |
| Ο  Never  (Go to Q5) | Ο  1-3 times per **month** | Ο  1-2 times per **week** | | Ο  3-4 times per **week** | Ο  5-6 times per **week** | Ο  1 time per **day** | Ο  2 times per **day** | Ο  3 times per **day** | | Ο  4 times per **day** | Ο  5 or more times/**day** |
| **4a.** Each time you ate **French fries** or **fried potatoes**, how much did you usually eat? | | | | | | | | | | | |
| Ο  Small Order or Less  (About 1 cup or less) | | | Ο  Medium Order  (About 1½ cups) | | | Ο  Large order  (About 2 cups) | | | Ο  Supersize order or more  (About 3 cups or more) | | |
| **5.** **Over the last 3 months**, how often did you eat other white potatoes? Count **baked, boiled**, and **mashed potatoes**, **potato salad**, and **white potatoes that were not fried**. | | | | | | | | | | | |
| Ο  Never  (Go to Q6) | Ο  1-3 times per **month** | Ο  1-2 times per **week** | | Ο  3-4 times per **week** | Ο  5-6 times per **week** | Ο  1 time per **day** | Ο  2 times per **day** | Ο  3 times per **day** | | Ο  4 times per **day** | Ο  5 or more times/**day** |
| **5a.** Each time you ate **these potatoes**, how much did you usually eat? | | | | | | | | | | | |
| Ο  1 small potato or less  (½ cup or less) | | | Ο  1 medium potato  (½ to 1 cup) | | | Ο  1 large potato  (1 to 1½ cups) | | | Ο  2 medium potatoes or more  (1½ cups or more) | | |
| **6.** **Over the last 3 months**, how often did you eat **cooked dried beans**? Count **baked beans, bean soup, refried beans, pork and beans**, and **other bean dishes**. | | | | | | | | | | | |
| Ο  Never  (Go to Q7) | Ο  1-3 times per **month** | Ο  1-2 times per **week** | | Ο  3-4 times per **week** | Ο  5-6 times per **week** | Ο  1 time per **day** | Ο  2 times per **day** | Ο  3 times per **day** | | Ο  4 times per **day** | Ο  5 or more times/**day** |
| **6a**. Each time you ate **these beans**, how much did you usually eat? | | | | | | | | | | | |
| Ο  Less than ½ cup | | | Ο  ½ to 1 cup | | | Ο  1 to 1½ cups | | | Ο  More than 1½ cups | | |

| **7.** **Over the last 3 months**, how often did you eat **other vegetables**? | | | | | | | | | | | | | | | | | | | | | | |
| --- | --- | --- | --- | --- | --- | --- | --- | --- | --- | --- | --- | --- | --- | --- | --- | --- | --- | --- | --- | --- | --- | --- |
| DO NOT COUNT:   - Lettuce salads - White potatoes - Cooked dried beans - Vegetables in mixtures, such as sandwiches, omelets, Mexican dishes, stews, stir-fries, soups, etc. - Rice   COUNT:   - All other vegetables – raw, cooked, canned, and frozen | | | | | | | | | | | | | | | | | | | | | | |
| Ο  Never  (Go to Q8) | Ο  1-3 times per **month** | | Ο  1-2 times per **week** | | | | Ο  3-4 times per **week** | | Ο  5-6 times per **week** | | Ο  1 time per **day** | | Ο  2 times per **day** | | Ο  3 times per **day** | | | | Ο  4 times per **day** | | Ο  5 or more times/**day** | |
| **7a.** Each time you ate **other vegetables**, how much did you usually eat? | | | | | | | | | | | | | | | | | | | | | | |
| Ο  Less than ½ cup | | | | | Ο  ½ to 1 cup | | | | | | Ο  1 to 1½ cups | | | | | | Ο  More than 1½ cups | | | | | |
| **8.** **Over the last 3 months**, how often did you eat **tomato sauce**? Include tomato sauce on pasta or   macaroni, rice, pizza and other dishes. | | | | | | | | | | | | | | | | | | | | | | |
| Ο  Never  (Go to Q9) | Ο  1-3 times per **month** | | Ο  1-2 times per **week** | | | | Ο  3-4 times per **week** | | Ο  5-6 times per **week** | | Ο  1 time per **day** | | Ο  2 times per **day** | | Ο  3 times per **day** | | | | Ο  4 times per **day** | | Ο  5 or more times/**day** | |
| **8a.** Each time you ate **tomato sauce**, how much did you usually eat? | | | | | | | | | | | | | | | | | | | | | | |
| Ο  About ¼ cup | | | | | Ο  About ½ cup | | | | | | Ο  About 1 cup | | | | | | Ο  More than 1 cup | | | | | |
| **9.** **Over the last 3 months**, how often did you eat **vegetable soups**? Include tomato soup, gazpacho,   beef with vegetable soup, minestrone soup, and other soups made with vegetables. | | | | | | | | | | | | | | | | | | | | | | |
| Ο  Never  (Go to Q10) | Ο  1-3 times per **month** | | Ο  1-2 times per **week** | | | | Ο  3-4 times per **week** | | Ο  5-6 times per **week** | | Ο  1 time per **day** | | Ο  2 times per **day** | | Ο  3 times per **day** | | | | Ο  4 times per **day** | | Ο  5 or more times/**day** | |
| **9a.** Each time you ate **vegetable soup**, how much did you usually eat? | | | | | | | | | | | | | | | | | | | | | | |
| Ο  Less than 1 cup | | | | | | Ο  1 to 2 cups | | | | | | Ο  2 to 3 cups | | | | | | Ο  More than 3 cups | | | | |
| **10.** **Over the last 3 months**, how often did you eat **mixtures that included vegetables**? Count such foods as sandwiches, casseroles, stews, stir-fries, omelets, and tacos. | | | | | | | | | | | | | | | | | | | | | | |
| Ο  Never | | Ο  1-3 times per **month** | | Ο  1-2 times per **week** | | | | Ο  3-4 times per **week** | | Ο  5-6 times per **week** | | Ο  1 time per **day** | | Ο  2 times per **day** | | Ο  3 times per **day** | | | | Ο  4 times per **day** | | Ο  5 or more times/**day** |

**This section is about your health and physical activity habits, answer each question to the best of your ability.**

1. Please respond to each item by marking one box per row:

|  | **Poor** | **Fair** | **Good** | **Very Good** | **Excellent** |
| --- | --- | --- | --- | --- | --- |
| In general, would you say that your health is: |  |  |  |  |  |
| In general, would you say that your quality of life is: |  |  |  |  |  |
| In general, how would you rate your physical health? |  |  |  |  |  |
| In general, how would you rate your mental health, including your mood and your ability to think? |  |  |  |  |  |
| In general, how would you rate your satisfaction with your social activities and relationships? |  |  |  |  |  |

1. To what extent are you able to carry out your everyday physical activities such as walking,
   climbing stairs, carrying groceries, or moving a chair?

| - Not at all able | - A little able | - Moderately able | - Mostly able | - Completely able |
| --- | --- | --- | --- | --- |

1. In the past 3 months, how would you rate your pain on average? (Circle your answer)

**No pain** 1 2 3 4 5 6 7 8 9 10 **Worst pain imaginable**

1. In the past 3 months, how would you rate your fatigue on average?

| - None | - Mild | - Moderate | - Severe | - Very Severe |
| --- | --- | --- | --- | --- |

1. In the past 3 months, in general, please rate how well you carry out your usual social activities and roles. (This includes activities at home, at work and in your community, and responsibilities as a parent, child, spouse, employee, friend, etc.)

| - Poor | - Fair | - Good | - Very Good | - Excellent |
| --- | --- | --- | --- | --- |

1. In the past 3 months, how often have you been bothered by emotional problems such as feeling anxious, depressed or irritable?

| - Never | - Rarely | - Sometimes | - Often | - Always |
| --- | --- | --- | --- | --- |

1. Think about your use of the following products over the **last 3 months**. About how often did you consume each of the following? Mark only one frequency selection on each row.

|  | **Never** | **Less than once** | **1-3 times** | **1-2 times per week** | **3-4 times per week** | **5-6 times per week** | **Once per day** | **2 times per day** | **3-4 times per day** | **5+ times per day** |
| --- | --- | --- | --- | --- | --- | --- | --- | --- | --- | --- |
| Coffee | 🞎 | 🞎 | 🞎 | 🞎 | 🞎 | 🞎 | 🞎 | 🞎 | 🞎 | 🞎 |
| Tea | 🞎 | 🞎 | 🞎 | 🞎 | 🞎 | 🞎 | 🞎 | 🞎 | 🞎 | 🞎 |
| Caffeinated soft drinks, energy drinks  (i.e., Coke, Redbull) | 🞎 | 🞎 | 🞎 | 🞎 | 🞎 | 🞎 | 🞎 | 🞎 | 🞎 | 🞎 |
| Alcohol | 🞎 | 🞎 | 🞎 | 🞎 | 🞎 | 🞎 | 🞎 | 🞎 | 🞎 | 🞎 |
| Aspirin | 🞎 | 🞎 | 🞎 | 🞎 | 🞎 | 🞎 | 🞎 | 🞎 | 🞎 | 🞎 |
| Over the counter pain relievers | 🞎 | 🞎 | 🞎 | 🞎 | 🞎 | 🞎 | 🞎 | 🞎 | 🞎 | 🞎 |
| Cold medications | 🞎 | 🞎 | 🞎 | 🞎 | 🞎 | 🞎 | 🞎 | 🞎 | 🞎 | 🞎 |
| Allergy medicine/ antihistamines | 🞎 | 🞎 | 🞎 | 🞎 | 🞎 | 🞎 | 🞎 | 🞎 | 🞎 | 🞎 |
| Sleeping pills/tranquilizers | 🞎 | 🞎 | 🞎 | 🞎 | 🞎 | 🞎 | 🞎 | 🞎 | 🞎 | 🞎 |

1. Please answer the next four questions about physical activity as they have applied to you over the **past 3 months:**

**8a.** How many days per week did you exercise or take part in **vigorous** physical activities that made you sweat and breathe hard for at least 30 minutes (such as basketball, jogging, swimming laps, tennis, fast bicycling, strength training, or similar)?

Please circle one: 0 1 2 3 4 5 6 7

**8b.** How many days per week did you exercise or take part in **moderate** physical activities that increased your breathing for a total of at least 30 minutes (such as brisk walking)? *Do not include activities from question above.*

Please circle one: 0 1 2 3 4 5 6 7

**8c.** How many days per week did you exercise to **strengthen or tone** your muscles (such as push-ups, sit-ups, or weight lifting)? *This can include activities you counted in the above questions.*

Please circle one: 0 1 2 3 4 5 6 7

**8d.** In a typical week, how many days do you take part in any physical activity long enough to work up a sweat? *This can include activities counted in questions above.*

Please circle one: 0 1 2 3 4 5 6 7

1. **How would you describe your typical day in the last 3 months?**

__________ % Sitting (including driving)

__________ % Standing

__________ % Walking

__________ % Heavy labor or physically demanding tasks

=100% total

**This is the last section.**

**You are almost done!**

**1.** Please note each major life event that you have experienced in the last 3 months:

| **Life Event** | **Yes** | **No** |
| --- | --- | --- |
| Death of spouse |  |  |
| Divorce |  |  |
| Marital separation from mate |  |  |
| Detention in jail or other institution |  |  |
| Death of a close family member |  |  |
| Major personal injury or illness |  |  |
| Marriage |  |  |
| Being fired at work |  |  |
| Marital reconciliation with mate |  |  |
| Retirement from work |  |  |
| Major change in health or behavior of a family member |  |  |
| Pregnancy |  |  |
| Sexual difficulties |  |  |
| Gaining a new family member (i.e. birth, adoption, older adult moving in, etc.) |  |  |
| Major business readjustment |  |  |
| Major change in financial state (i.e. a lot worse or better off than usual) |  |  |
| Death of a close friend |  |  |
| Changing to a different line of work |  |  |
| Major change in the number of arguments w/spouse (i.e. either a lot more or a lot less than usual regarding child rearing, personal habits, etc.) |  |  |
| Taking on a mortgage |  |  |
| Foreclosure on a mortgage or loan |  |  |
| Major change in responsibilities at work (i.e. promotion, demotion, etc.) |  |  |
| Son or daughter leaving home (marriage, attending college, joined military) |  |  |
| In-law troubles |  |  |
| Outstanding personal achievement |  |  |
| Spouse beginning or ceasing work outside the home |  |  |
| Beginning or ceasing formal schooling |  |  |
| Major change in living condition (new home, remodeling, deterioration of neighborhood or home etc.) |  |  |
| Revision of personal habits (dress manners, associations, quitting smoking) |  |  |
| Troubles with the boss |  |  |
| Major changes in working hours or conditions |  |  |
| Changes in residence |  |  |
| Changing to a new school |  |  |
| Major change in usual type and/or amount of recreation |  |  |
| Major change in church activity (i.e. a lot more or less than usual) |  |  |
| Major change in social activities (clubs, movies, visiting, etc.) |  |  |
| Taking on a loan (car, TV, freezer, etc.) |  |  |
| Major change in sleeping habits (a lot more or less than usual) |  |  |
| Major change in number of family get-togethers (a lot more or less than normal) |  |  |
| Major change in eating habits (a lot more or less intake, or very different meal hours or surroundings) |  |  |
| Vacation |  |  |
| Major holidays |  |  |
| Minor violations of the law (traffic tickets, jaywalking, disturbing the peace, etc.) |  |  |

**Thank you for taking this survey!**

**Please alert the research staff when you are done.**
